# Numerical Simulation and Experimental Study of Fluid-Solid Coupling-Based Air-Coupled Ultrasonic Detection of Stomata Defect of Lithium-Ion Battery

**DOI:** 10.3390/s19102391

**Published:** 2019-05-25

**Authors:** Honggang Li, Zhenggan Zhou

**Affiliations:** 1 School of Mechanical Engineering and Automation, Beihang University, Beijing 100083, China; zzhenggan@buaa.edu.cn; 2 The Collaborative Innovation Center for Advanced Aero-Engine (CICAAE), Beijing 100083, China

**Keywords:** fluid–solid coupling, air-coupled ultrasonic testing, numerical simulation, lithium-ion battery

## Abstract

Aiming at the characteristics of the periodic stacking structure of a lithium-ion battery core and the corresponding relationship between the air-coupled ultrasonic transmission initial wave and the wave propagation mode in each layer medium of a lithium-ion battery, the homogenized finite element model of a lithium-ion battery was developed based on the theory of pressure acoustics and solid mechanics. This model provided a reliable method and basis for solving the visualization of ultrasonic propagation in a lithium-ion battery and the analysis of ultrasonic time-frequency domain characteristics. The finite element simulation analysis and experimental verification of a lithium-ion battery with a near-surface stomata defect, near-bottom stomata defect and middle-layer stomata defect were performed. The results showed that the air-coupled ultrasonic transmission signal can effectively characterize the stomata defect inside a lithium-ion battery. The energy of an air-coupled ultrasonic transmission signal is concentrated between 350–450 kHz, and the acoustic diffraction effect has an important influence on the effect of the ultrasonic and stomata defect. Based on the amplitude response characteristics of the air-coupled ultrasonic transmission wave in the stomata defect area, a C-scan of the lithium-ion battery was performed. The C-scan result verified that air-coupled ultrasonic testing technology can accurately and effectively detect the pre-embedded stomata defect and natural stomata defect in a lithium-ion battery, which is able to promote and expand the application of the technology in the field of electric energy security.

## 1. Introduction

In recent years, a lithium-ion battery has been widely used in energy transportation, aerospace and military field because of its high energy density, high operating voltage, little self-discharge rate, long cycle life and environmental friendliness [1,2,3,4,5,6,7]. Therefore, a lithium-ion battery is considered to be the most promising high-efficiency power reserve device. Due to the particularity of the preparation process and service environment, pole piece misalignment, stomata, burr and other defects often appear in the end product and these defects may result in a short-circuit or leakage, which will further cause thermal runaway, combustion and explosion of the lithium-ion battery [8,9,10,11]. This seriously affects the security of the service environment. A stomata defect is formed in the actual service process. It usually refers to a variety of gases that are generated by a large amount of decomposition of the lithium-ion battery internal substances due to internal abnormal behavior (excessive moisture content, loss of solid electroly interphase film, etc.) of abnormal environment work (overcharge, overdischarge, high temperature environment, etc.) after the air-big is removed and package is finished [12,13]. The internal gas components of the stomata defect mainly include CO_2_, CO, alkane gas, O_2_, H_2_ and so on [14,15,16,17]. In order to avoid such defects affecting the safety and reliability of the lithium-ion battery, in addition to strict control of the preparation process and development of an effective battery management system, a reliable and effective non-destructive testing method must be provided to eliminate short-board batteries. Furthermore, the consistency of each single-cell battery in a lithium-ion battery pack is guaranteed. The performance and safety of the lithium-ion battery pack is also guaranteed. 

At present, there are ultrasonic testing technology and X-ray technology for the detection of the stomata defect of a lithium-ion battery [18,19]. However, the couplant (water, coupling oil, etc.) used in traditional ultrasonic testing technology will cause damage and pollution to the lithium-ion battery cell, and its detection efficiency is very low. X-ray technology can identify such a defect, but this technology will cause harm to the service environment of the lithium-ion battery due to radiation, and it is difficult to adapt to on-site testing. Therefore, it is necessary to develop a non-contact, completely non-destructive, non-coupling agent and have less impact on service environment testing technology. 

The air-coupled ultrasonic testing (ACUT) technology is a new non-destructive ultrasonic testing technology. This technology uses air as a coupling medium and has several significant advantages: Non-contact, completely nondestructive [20], strong defect identification and characterization, high detection efficiency [21,22], etc. Moreover, this technology can realize automatic detection of large-scale and complex components, and has excellent on-site detection capability. Therefore, it is very promising to apply this technology to a quality evaluation of a lithium-ion battery.

This paper studies and demonstrates the application of air-coupled ultrasonic testing technology to nondestructive testing of the stomata defect in a lithium-ion battery. Firstly, based on the characteristics of the periodic stacking structure of a lithium-ion battery core and the corresponding relationship between the air-coupled ultrasonic transmission initial wave and the wave propagation mode in each layer medium of the lithium-ion battery, a modeling method for the homogenized finite element model of a lithium-ion battery is first proposed. Based on the theory of pressure acoustics and solid mechanics, the wave equations of the fluid–solid coupling system are deduced, the boundary of the acoustic-structure is established, and visualization of the transmission of the ultrasonic wave in a lithium-ion battery is realized. Secondly, in order to simulate the presence of a near-surface stomata defect, near-bottom stomata defect and middle-layer stomata defect of a lithium-ion battery, air layers are respectively set up in the first to sixth electrolyte layers in the homogenized finite element model of a lithium-ion battery. The time-frequency domain analysis method is used to analyze the effect of the acoustic wave and stomata defect in the electrolyte of each layer at different transient moments. It has been proved that a simulated transmission signal can effectively characterize the stomata defect in the electrolyte of each layer. Finally, the C-scan detection is performed on a lithium-ion battery with embedded defects based on the transmitted initial wave of an air-coupled ultrasonic signal. The accuracy and effectiveness of the air-coupled ultrasonic testing method is verified.

## 2. Theory and Model

**Figure 1:**
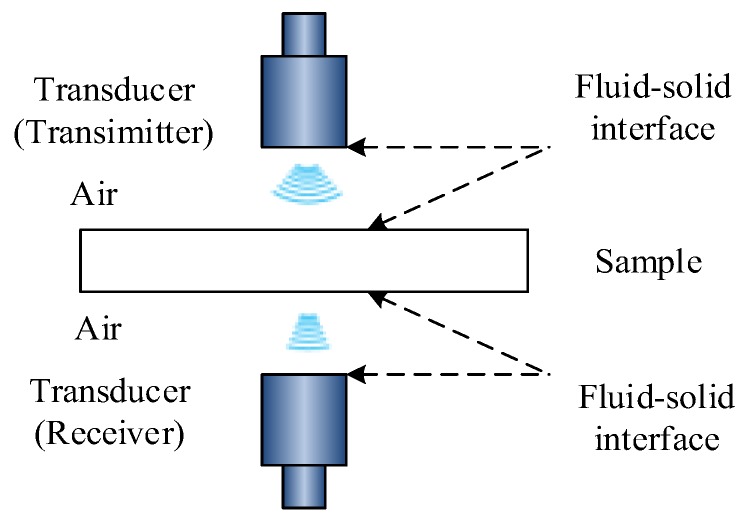
The schematic diagram of an ultrasound transmission.

Under air-coupled conditions, an ultrasonic wave is excited, propagated and received through a variety of media and fluid–solid coupling interfaces, particularly in air-coupled ultrasonic testing of a lithium-ion battery. For a lithium-ion battery, the problem of ultrasonic propagation in air and electrolyte media can be attributed to the pressure acoustics problem in the fluid field. The problem of ultrasonic propagation in aluminum, copper, graphite, polypropylene (PP), nylon, etc. can be attributed to the stress-strain problem in the solid field. The problem of ultrasonic propagation at the fluid–solid coupling interface can be attributed to the fluid–solid coupling stress-strain problem under certain continuous conditions. Therefore, it is necessary to analyze and discuss the wave equation in fluid mechanics, structural mechanics equation, fluid–solid dynamic coupling equation and boundary conditions to provide accurate and reliable theoretical basis for finite element analysis. The schematic diagram of ultrasound transmission is shown in Figure 1.

### 2.1. Finite Element Method

Pressure loads are applied in air and electrolyte domains. Displacement acceleration of particles in the air and electrolyte domains are caused by an external disturbance, and propagates forward in a continuous form. The propagation of a sound wave in the fluid domain can be described by Equation (1):

(1) $$\left[M_{f}\right]\left\{\ddot{P_{f}}\right\}+\left[K_{f}\right]\left\{P_{f}\right\}+\rho_{0}{\left[R_{f}\right]}^{T}\left\{\ddot{u_{f}}\right\}=0$$

where $\left[M_{f}\right]$ is the mass matrix of the fluid medium, $\left[K_{f}\right]$ is the stiffness matrix of the fluid medium, $\;\left\{P_{f}\right\}$ is the acoustic pressure vector of the element node in the fluid medium, $\;\left\{\ddot{u_{f}}\right\}$ is the acceleration vector of the element node in the fluid medium.

The propagation of a sound wave in the solid field, such as, aluminum, copper, graphite, polypropylene polymer, nylon, etc., can be described by formula (2):

(2) $$\left[M_{s}\right]\left\{\ddot{U}\right\}+\left[K_{s}\right]\left\{U\right\}=\left\{F_{s}\right\}$$

where $\left[M_{s}\right]$ is the mass matrix of the solid medium, $\left\{F_{s}\right\}$ is the pressure load of the solid medium, $\;\left[K_{s}\right]$ is the stiffness matrix of the solid medium, {U} is the displacement vector of the solid medium.

When a sound wave reaches the fluid–solid coupling interface, the dynamic interaction between the fluid medium at the fluid interface and the solid medium at the solid interface need to be considered. Therefore, the sound wave control equation of the fluid–solid coupling interface is [23]:

(3) $$\left[\begin{array}{c} \rho_{0}R_{f}^{T}\;\left[M_{f}\right]\\ \left[M_{s}\right]\;\;\;\;\;0\;\; \end{array}\right]\left\{\begin{array}{c} \left\{\ddot{U}\right\}\\ \left\{\ddot{P_{f}}\right\} \end{array}\right\}+\left[\begin{array}{c} 0\;\;\;\;\left[K_{f}\right]\\ \left[K_{s}\right]\;-R]\;\; \end{array}\right]\left\{\begin{array}{c} \left\{U\right\}\\ \left\{P_{f}\right\} \end{array}\right\}=\left\{\begin{array}{c} \left\{F_{f}\right\}\\ \left\{F_{s}\right\} \end{array}\right\}$$

where [R] is the coupling matrix at the fluid-solid coupling interface. This equation means that the nodes at the fluid–solid interface are continuous and the nodes both have a displacement component and pressure component.

### 2.2. Finite Element Model

#### 2.2.1. The Development Model

**Figure 2:**
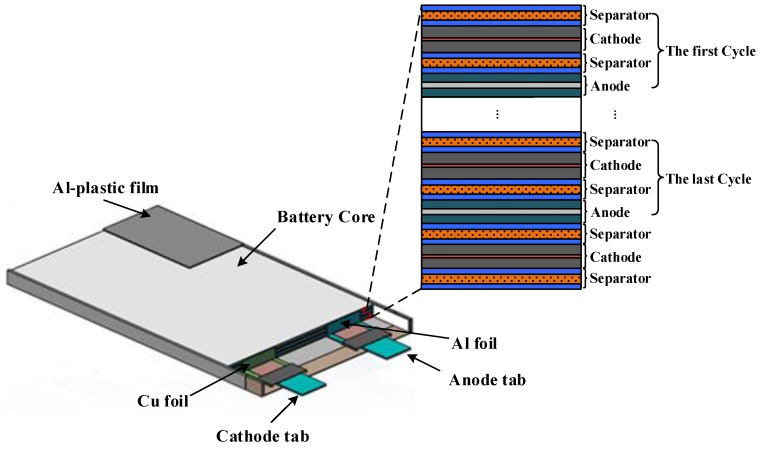
The composition of a lithium-ion battery.

**Figure 3:**
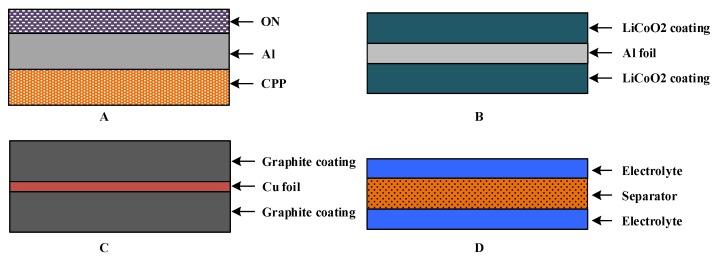
The schematic diagram of the composition unit structure of a lithium-ion battery (**A**) The schematic diagram of Al-plastic film; (**B**) The schematic diagram of anode electrode plate; (**C**) The schematic diagram of cathode electrode plate; (**D**) The schematic diagram of separator.

**Figure 4:**
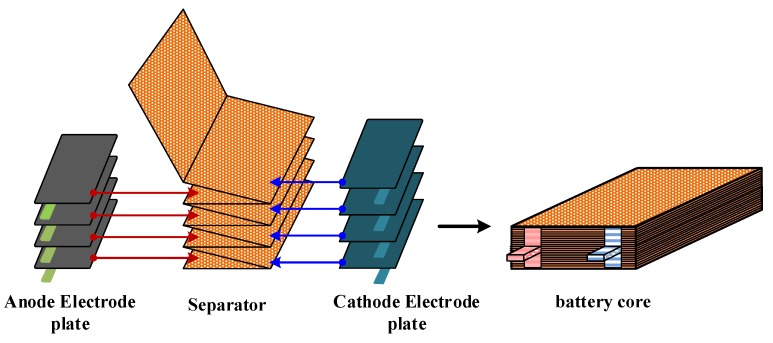
The schematic diagram of the battery core structure.

A lithium-ion battery consists of an Al-plastic film, battery core, current collector and battery tab [24,25], as shown in Figure 2. An Al-plastic film is composed of nylon, polypropylene and thin aluminum foil, and the schematic diagram is shown in Figure 3A. The battery core is a multilayer laminated composite composed of an anode electrode plate, separator, cathode electrode plate and electrolyte, as shown in Figure 4. The anode electrode plate consists of two layers of positive electrode active materials (LiCoO2 coating) and one layer of the metal current collector (Al foil), as shown in Figure 3B. The cathode electrode plate consists of two layers of negative electrode active materials (graphite coating) and one layer of the metal current collector (Cu foil), as shown in Figure 3C. The separator consists of two layers of electrolyte and one layer of the polypropylene polymer material (PP), as shown in Figure 3D. 

The lithium-ion battery used in this paper consisted of 10 layers of anode electrode plates (three-layer structure), 11 layers of cathode electrode plates (three-layer structure), 22 layers of separators and two layers of Al-plastic film (three-layer structure). The total number layers of the lithium-ion battery cell were up to 135 layers, and each layer was in the micron scale. Therefore, the establishment of the finite element model for a lithium-ion battery has many problems, such as difficulty in model establishment, difficulty in grid division and a huge calculation amount.

**Figure 5:**
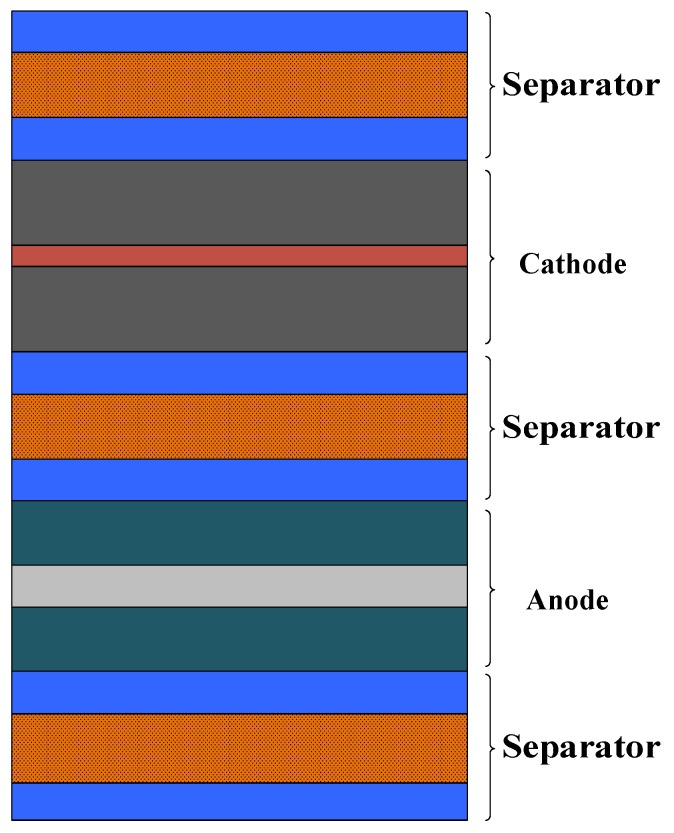
The homogenization model of the lithium-ion battery core.

In order to solve the above problems, after carefully observing the structure of the lithium-ion battery (Figure 2, Figure 3 and Figure 4), it was found that the structure of lithium-ion battery core has a periodic distribution characteristic. The single cycle was arranged in the form of a separator–cathode–separator–anode, and finally ended with a single cycle of a missing anode electrode plate (separator–cathode–separator). Therefore, According to the characteristic of the periodic structure of the lithium-ion battery core (Figure 2), a simplified model of the lithium-ion battery was established, that is, the mediums that periodically appear were integrated together, and the thickness of mediums was enlarged in proportion (this model was named as the homogenization model of the lithium-ion battery). Then, the structure of the battery core model after homogenization was as follows: Separator–cathode–separator–anode–separator (taking into account the actual structure of the lithium-ion battery, the separator needs three homogeneous treatments), as shown in Figure 5. After the homogenization process, the layer number of the medium of the lithium-ion battery was reduced from 135 layers to 21 layers, and the inner interface of the lithium-ion battery was reduced from 134 to 20. In this case, the difficulty of modeling was greatly reduced and the mesh was reasonable.

In order to verify the rationality of the homogenization model, it was necessary to clarify the attenuation factor of the sound wave propagating inside the battery and the reference sound wave mode for actual detection. For the sound wave propagating inside the lithium-ion battery, the main factors causing attenuation include absorption attenuation of medium, scattering attenuation of medium, diffusion attenuation and interface attenuation [26,27,28]. Among them, the influence of interface attenuation is the most prominent. Therefore, the effect of interface attenuation was mainly analyzed. The acoustic impedance of the medium is the inherent physical property of the medium. When the sound wave passes though the same interface, the attenuation energy ratio is the same, as shown in formula (4), *Z_i* is the acoustic impedance of the medium *i* (*i* = 1,2).

(4) $$T_{12}=\frac{2Z_{2}}{Z_{1}+Z_{2}}$$

(5) $$Z_{i}=\rho_{i}c_{i},i=1,2$$

Therefore, when the homogenization model is used, the significant reduction of the interfacial layer number of medium of lithium-ion will cause echo signal energy to be amplified by a certain coefficient. Furthermore, when ACUT technology is used to actually detect the lithium-ion battery, the reference sound wave is the transmission initial wave (corresponding to the longitudinal wave with the fastest propagation speed in each medium of the lithium-ion battery). However, the reduction of the interfacial layer number of the medium has little effect on the mode and time of flight (TOF) of the transmission initial wave (Snell theorem). Therefore, acoustic simulation of the lithium-ion battery based on the homogenization model is reasonable.

**Figure 6:**
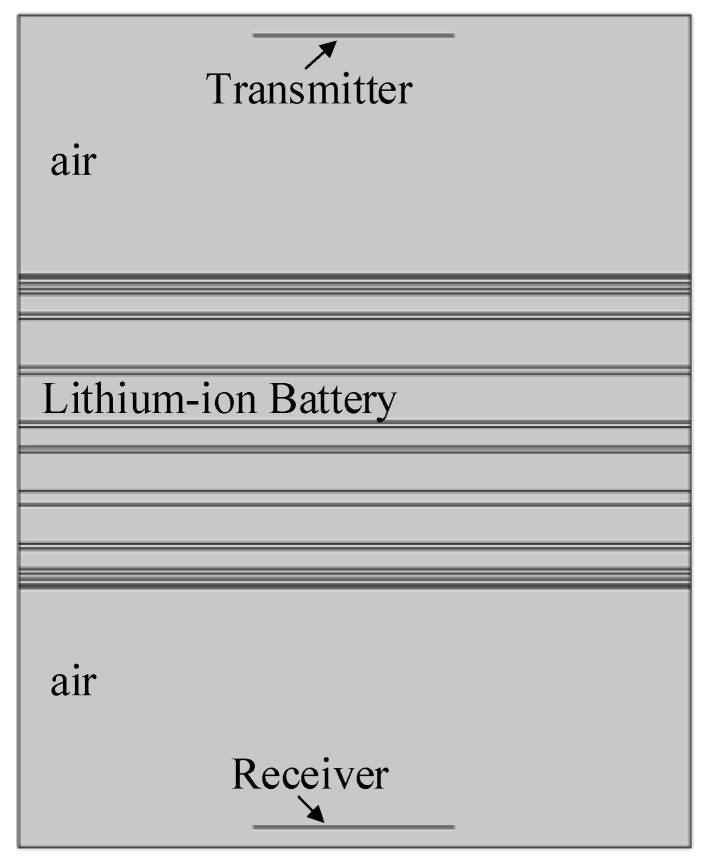
The homogenization finite element model of the lithium-ion battery.

In order to maximize the energy of the sound source coupled into the lithium-ion battery, the distance between sound source and lithium-ion battery was set as the focal length of the sound source. The homogenization finite element model of the lithium-ion battery is shown in Figure 6. The model includes the air domain, lithium-ion battery domain and radiation sound source.

#### 2.2.2. Stomata Defect Positing Setting in the Lithium-Ion Battery Model

**Figure 7:**
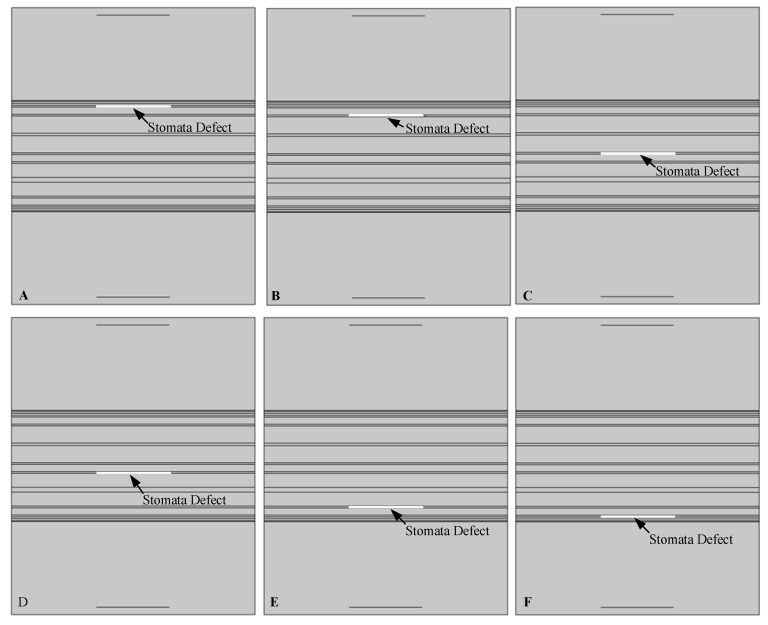
The defect configuration in lithium-ion battery model (**A**) First electrolyte layer; (**B**) Second electrolyte layer; (**C**) Third electrolyte layer; (**D**) Fourth electrolyte layer; (**E**) Fifth electrolyte layer; (**F**) Sixth electrolyte layer.

In order to study the response of the ultrasonic wave to the stomata defect in the lithium-ion battery, air layers were embedded in the electrolyte layers from the first to the sixth of the lithium-ion battery (simulating the stomata defect inside the battery), as shown in Figure 7. The first layer and the second layer represented the near surface layer of the lithium-ion battery, the third layer and the fourth layer represented the middle layer of the lithium-ion battery and the fifth layer and sixth layer represented near the bottom layer of the lithium-ion battery.

#### 2.2.3. Finite Element Mesh and Time Step

In order to accurately invert the propagation of the sound wave in each layer of the lithium-ion battery, and taking into account the amount of finite element calculation, mesh size division was very important. In this paper, different mesh sizes were selected for different mediums. Usually, 1/10 of a wavelength of the sound wave in the current medium was used as the mesh size, as shown in Formula (13), where C is the sound velocity of medium and f is the frequency of sound wave. The result of meshing is shown in Table 1. Obviously, the meshing results meet Formula (6):

(6) $$L_{mesh}\leq \frac{1}{10}\frac{C}{f}$$

In order to fully display the details of the propagation of the sound wave, time step was set to 18 ns.

#### 2.2.4. Boundary Condition and Material Property

Reflection echo has a great influence on the result of the finite element analysis. In order to avoid this situation, fluid domains (air domain and electrolyte domain) need to add the cylindrical wave radiation boundary. The formula is shown in Formula (7):

(7) $$-n\cdot \left(-\frac{1}{\rho }\left(\nabla P-q_{d}\right)\right)+\frac{1}{\rho }\left(\frac{1}{c}\frac{\partial P}{\partial t}+\frac{P}{2r}\right)=Q_{i}$$

where, *ρ* is the density of the fluid material, n is the unit normal vector of the fluid material from the inside to outside, $q_{d}$ is a dipole source, $Q_{i}$ is a unipolar source, P is the sound pressure of the fluid domain, *t* is time and *r* is the distance from the cylindrical wave radiation source. 

The boundary of the solid domain was selected with a low-reflection boundary, which can achieve a good absorption effect of the boundary sound wave. The formula is shown in Formula (8):

(8) $$\sigma \cdot n=-d_{i}\frac{\partial u}{\partial t}$$

where, *n* is the boundary direction vector, $d_{i}$ is the function of density ρ, shear wave velocity $C_{s}$ and longitudinal wave velocity $\;C_{p}$.

A line source composed of a discrete point was used as the transmitting transducer. The excitation signal was a cosine wave pulse treated with a Hanning window, and the amplitude of acoustic pressure was 100 Pa. The receiving transducer is completed by using the acoustic pressure integral of discrete points on the line source.

The linear elastic material was used in the fluid domain. The acoustic velocity is shown in Table 1 and the density is shown in Table 2. The material properties of solid domain are shown in Table 3.

## 3. Specimen and ACUT System

### 3.1. Specimen

**Figure 8:**
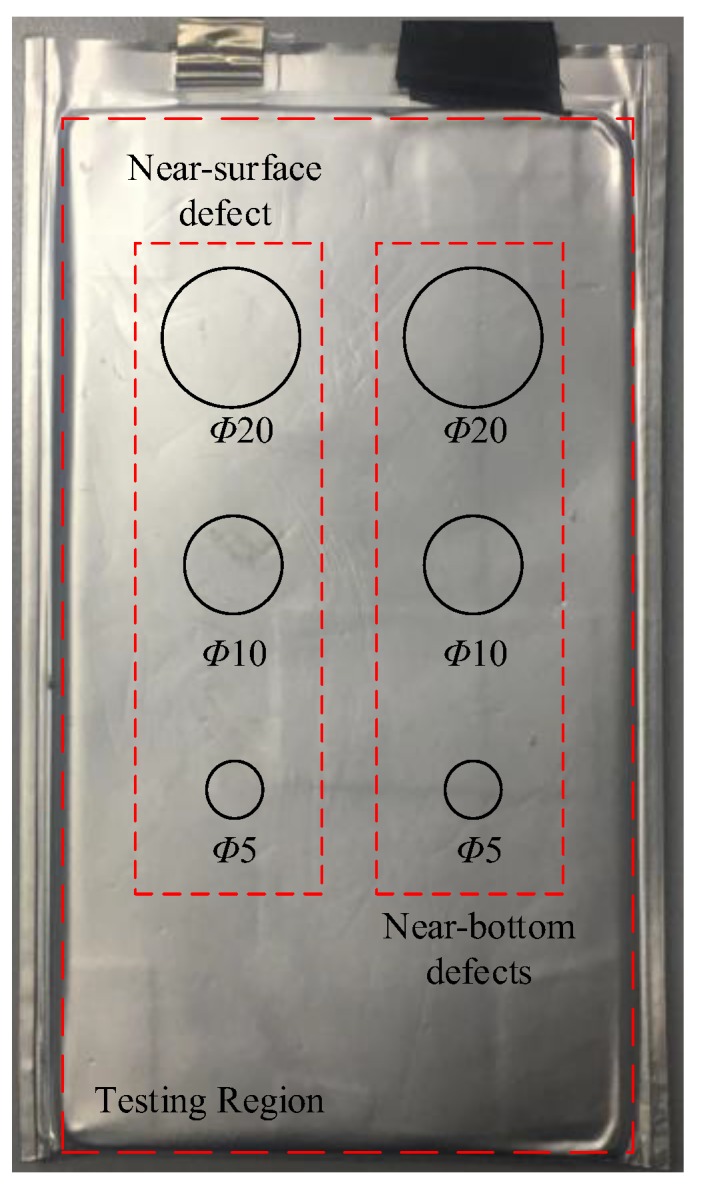
Honeycomb sandwich composite sample and defects layout.

The size of the lithium-ion battery sample was 160 mm × 80 mm × 45 mm. The voltage of the lithium-ion battery was 3.7 V. The capacity of the lithium-ion battery was 6000 mAh. The weight of the lithium-ion battery sample was 90 g. Three round copper flakes of Φ20 mm, Φ10 mm and Φ5 mm were pasted on the surface of the lithium-ion battery sample to simulate a near-surface stomata defect, and three round copper flakes of Φ20 mm, Φ10 mm and Φ5 mm were pasted on the bottom of the lithium-ion battery sample to simulate a near-bottom stomata defect, as shown in Figure 8.

### 3.2. ACUT System

**Figure 9:**
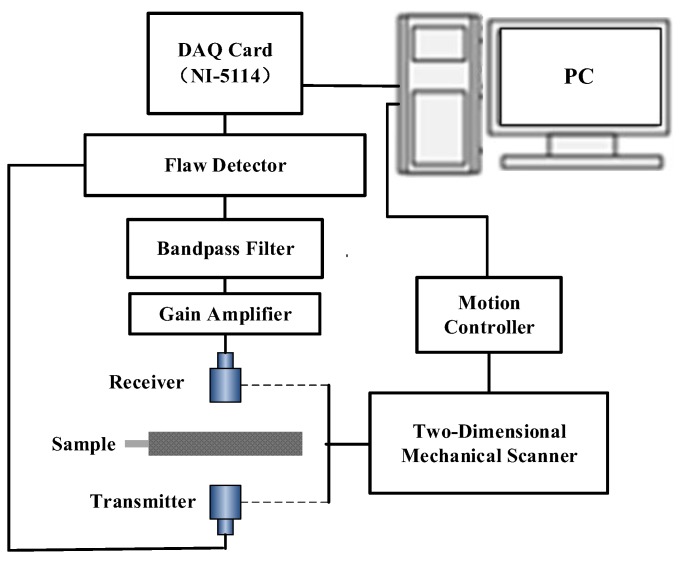
Schematic diagram of the experimental setup.

The air-coupled ultrasonic testing system was developed. The flaw detector (SONDA-007CX) was used to produce an exciting signal with a specific bandwidth and central frequency. Then, the signal was used to excite an air-coupled transmitting transducer to generate an ultrasonic signal. The ultrasonic signal passed through the lithium-ion battery sample and was received by the air-coupled receiving transducer. After being passed to the gain amplifier and bandpass filter device for gain compensation and filtering, the signal was again received by the flaw detector and finally collected by an 8 bits, 250 Msps ADC (NI PXI-5114, National instruments, Austin, TX, USA), which was embedded in the PC. Then, the collected data was transmitted to the PC for processing and an imaging operation. In conjunction with the mechanical scanning module, including mechanical two-dimensional scanning frame (LS-06), motion controller (MC-ST-02), servo motor, etc., the air-coupled ultrasonic testing system could perform a C-scan of the lithium-ion battery sample. The schematic diagram of the experimental setup is shown in Figure 9.

## 4. Results and Discussion

### 4.1. Ultrasonic Propagation Characteristic in the Lithium-Ion Battery

**Figure 10:**
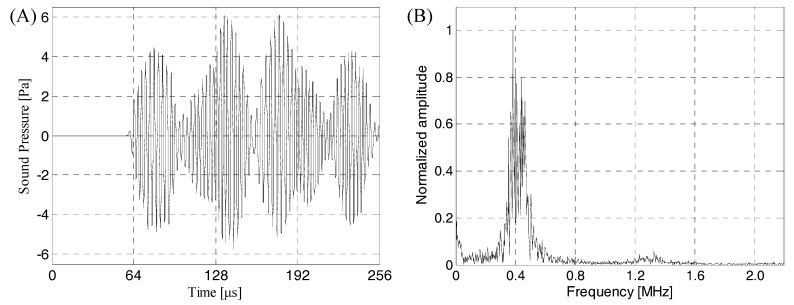
The time-frequency domain characteristics of the air-coupled ultrasonic signal passing through the lithium-ion battery. (**A**) Air-coupled ultrasonic time-domain signal passing through the lithium-ion battery; (**B**) the corresponding frequency spectrum.

The ultrasonic beam emitted by the air-coupled ultrasonic transducer was incident perpendicularly to the calculation area, and reflection, transmission and other acoustic phenomena occurred after contact with the lithium-ion battery sample. The sound pressure of the incident ultrasonic acted on the upper surface of lithium-ion battery, then it was converted to the internal stress of the lithium-ion battery and propagated downward in the form of stress. When the stress was transmitted to the interface between the lithium-ion battery and air, it was converted into sound pressure and radiated into the air domain. Then, it was received by the receiving transducer in the air domain. Figure 10 shows the received transmitted acoustic signal and its frequency spectrum.

As can be seen from Figure 10A, the finite element simulation time-domain signal had a good signal to noise ratio (SNR) and could clearly identify the transmitted initial wave signal. Therefore, it was speculated that the change of the energy amplitude of the transmitted initial wave could effectively evaluate and characterize the internal quality of the lithium-ion battery, and the transmitted initial wave was not easily interfered by other waveform signals. Figure 10B shows the received finite element simulation signal had narrow band characteristics. The signal energy was mainly concentrated in the 350–450 kHz. At about 400 kHz, the energy of the sound wave reached the maximum. Therefore, in actual detection, considering the penetration and resolution of the ACUT system and characteristics of the stomata defect (transverse size, longitudinal size, etc.), the air-coupled ultrasonic transducer with a center frequency of 400 kHz as the receiving transducer could achieve a good detection effect. And, in order to obtain accurate, reliable and easy-to-engineered air-coupled ultrasonic testing methods, we need to further study the ultrasonic response characteristics and characterization methods of the stomata defect at different positions in the lithium-ion battery sample.

### 4.2. Air-Coupled Ultrasonic Response and Characterization of the Stomata Defect of the Li-Ion Battery

On the basis of studying the time-frequency domain characteristic of an air-coupled transmission ultrasonic signal of the lithium-ion battery, the acoustic response of the stomata defect in the lithium-ion battery was further studied. It is the basis and premise for establishing the air-coupled ultrasonic characterization method of the stomata defect in the lithium-ion battery. The presence of the stomata defect in the internal electrolyte layer of the lithium-ion battery can cause a large number of sound waves to be reflected during sound wave transmission, and eventually cause the energy of air-coupled ultrasonic signal to be severely attenuated. Therefore, we could accurately evaluate the internal integrity of lithium-ion battery by measuring the attenuation of acoustic energy after penetrating the lithium-ion battery.

In order to accurately study the response of the stomata defect in each electrolyte layer of the lithium-ion battery to the ultrasonic signal, the first layer to the sixth layer of the electrolyte of the six battery models were respectively embedded with a stomata defect (air layer), as shown in Figure 7.

**Figure 11:**
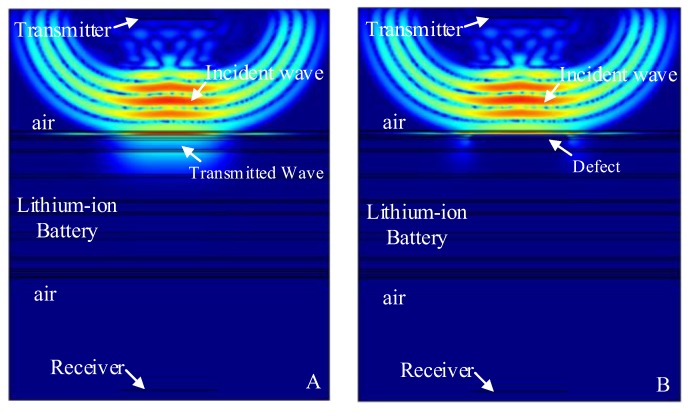
Finite element simulation of time snapshots of the propagation of the ultrasonic wave in the lithium-ion battery (11.268 μs). (**A**) The simulation result without a defect; (**B**) the simulation result with a stomata defect in the first electrolyte layer.

The finite element simulation comparison (11.268 μs) of the resultant stress amplitude of the intact lithium-ion battery and lithium-ion battery with the stomata defect in the first electrolyte layer (defect location as shown in Figure 7A) are shown in Figure 11. Figure 11A shows that the sound wave propagated in air and electrolyte mainly in the form of a longitudinal wave and the fastest propagation of the sound wave in the solid was the solid longitudinal wave. Since the ACUT mainly focuses on the effect of the transmission initial wave (corresponding to the longitudinal wave in air, liquid and solid) with a stomata defect, we focused on the effect of the longitudinal wave on defects in the lithium-ion battery. Figure 11B is the interaction between the longitudinal wave and stomata defect when the first electrolyte layer had a stomata defect: The energy of the transmission longitudinal wave attenuated significantly, and the amplitude energy was almost invisible in the simulation result. The results of the finite element analysis clearly showed an influence of the internal stomata defect of the lithium-ion battery on the propagation of the ultrasonic longitudinal wave. Therefore, it can be inferred that measuring the energy change of the transmission initial wave can effectively characterize the stomata defect in the first electrolyte layer of the lithium-ion battery.

**Figure 12:**
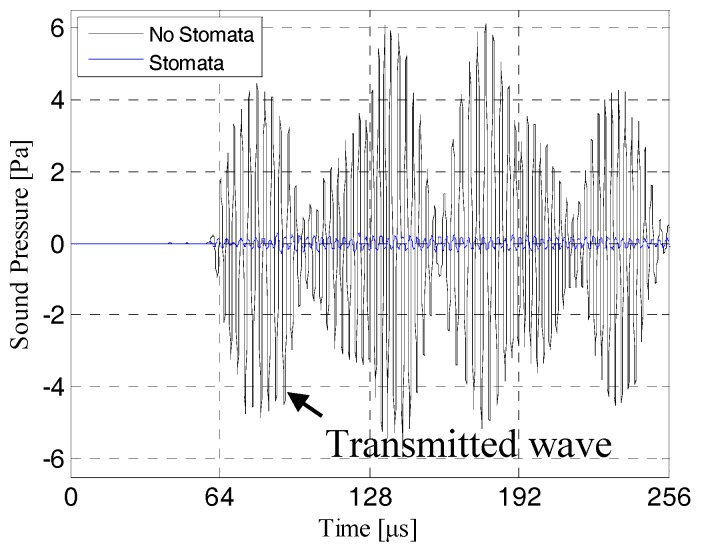
The transmission signals of the intact model and defective model with a stomata defect in the first electrolyte layer.

The contrast effect of the transmission ultrasonic signal received by the discrete probe points (corresponding to Figure 11) are shown in Figure 12. As can be seen from Figure 12A, when there was a stomata defect in the first electrolyte layer, the amplitude of transmission initial signal would be significantly attenuated, and the energy of the transmission initial signal was reduced from 4.449 Pa with no defect to 0.126 Pa with a stomata defect. Therefore, it could be confirmed that the stomata defects in the first electrolyte layer of the lithium-ion battery could be effectively characterized based on the change of the transmission initial wave.

**Figure 13:**
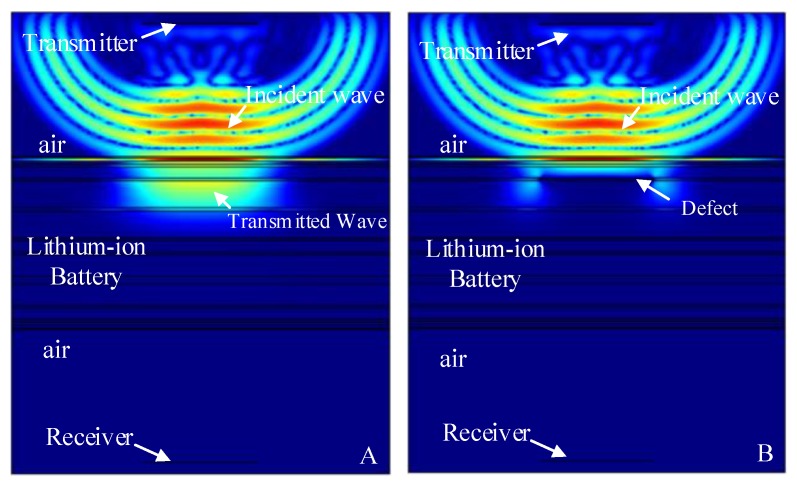
Finite element simulation of time snapshots of the propagation of the ultrasonic wave in the lithium-ion battery (11.520 μs). (**A**) The simulation result without a defect; (**B**) the simulation result with a stomata defect in the second electrolyte layer.

**Figure 14:**
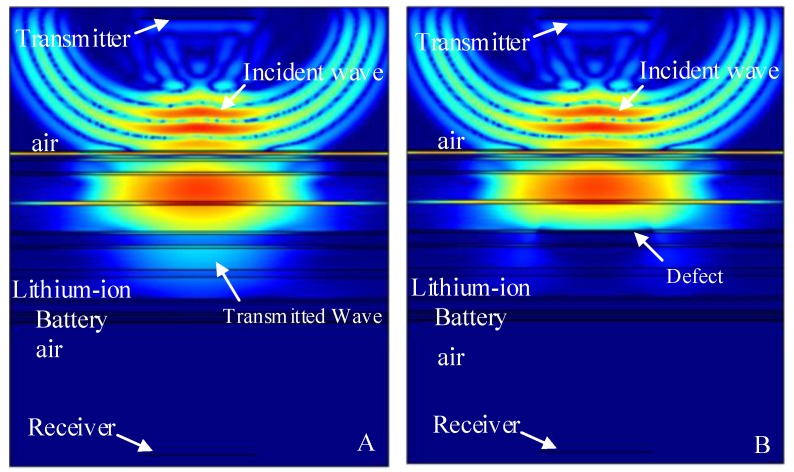
Finite element simulation of time snapshots of the propagation of the ultrasonic wave in the lithium-ion battery (12.060 μs). (**A**) The simulation result without a defect; (**B**) the simulation result with a stomata defect in the third electrolyte layer.

**Figure 15:**
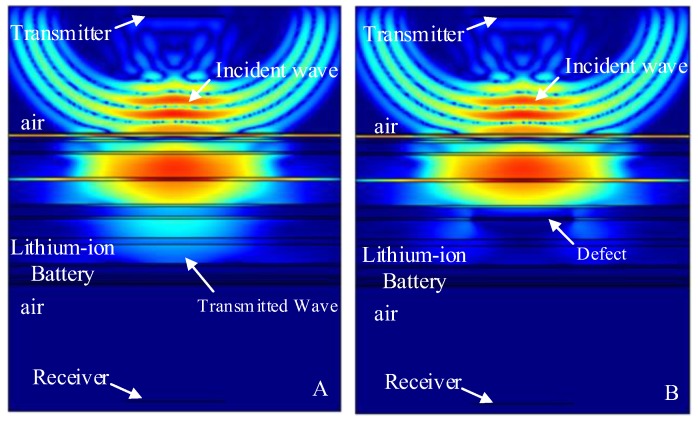
Finite element simulation of time snapshots of the propagation of the ultrasonic wave in the lithium-ion battery (12.186 μs). (**A**) The simulation result without a defect; (**B**) the simulation result with a stomata defect in the fourth electrolyte layer.

**Figure 16:**
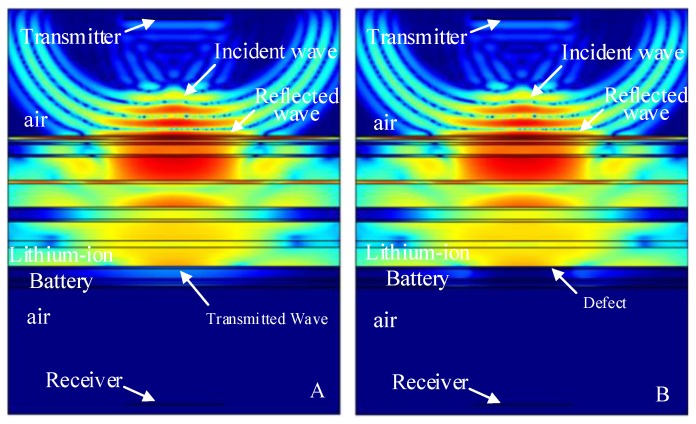
Finite element simulation of time snapshots of the propagation of the ultrasonic wave in the lithium-ion battery (12.996 μs). (**A**) The simulation result without a defect; (**B**) the simulation result with a stomata defect in the fifth electrolyte layer.

**Figure 17:**
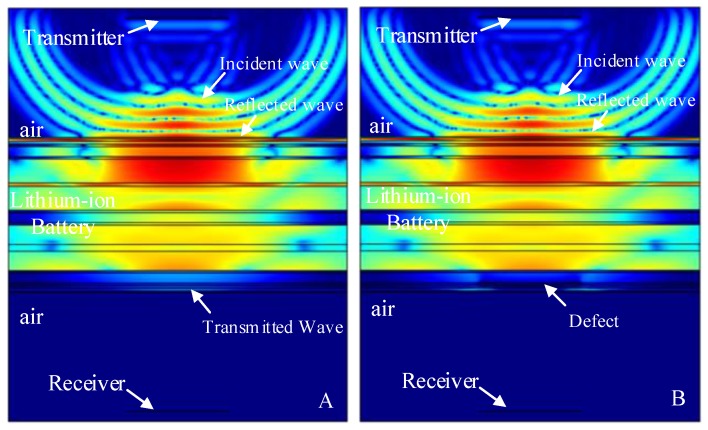
Finite element simulation of time snapshots of the propagation of the ultrasonic wave in the lithium-ion battery (13.122 μs). (**A**) The simulation result without a defect; (**B**) the simulation result with a stomata defect in the sixth electrolyte layer.

The finite element simulation comparison results (11.520 μs, 12.060 μs, 12.186 μs, 12.996 μs and 13.122 μs) of the intact lithium-ion battery and lithium-ion battery with a stomata defect in the second third, fourth, fifth and sixth electrolyte layer (defect location as shown in Figure 7) are shown in Figure 13, Figure 14, Figure 15, Figure 16 and Figure 17. Figure 13A, Figure 14A, Figure 15A, Figure 16A and Figure 17A show that the sound wave propagated in air and electrolyte mainly in the form of a longitudinal wave and the fastest propagation of the sound wave in the solid was the solid longitudinal wave. Since the ACUT mainly focuses on the effect of the transmission initial wave (corresponding to the longitudinal wave in air, liquid and solid) with stomata defects, we focused on the effect of the longitudinal wave and defect when the sound wave propagates inside the lithium-ion battery. Figure 13B, Figure 14B, Figure 15B, Figure 16B and Figure 17B show the interaction between the longitudinal wave and stomata defects (embedded in the second, third, fourth, fifth and sixth electrolyte layers of the lithium-ion battery): The energy of the transmission longitudinal wave attenuated significantly, the amplitude energy was almost invisible in the simulation result, the stress energy attenuation phenomenon occurred in the polypropylene area due to interference effect and there was a weak diffraction effect at the edge of the stomata defect. The results of the finite element analysis clearly showed an influence of the internal stomata defect of the lithium-ion battery on the propagation of the ultrasonic longitudinal wave. Therefore, it could be inferred that measuring the energy change of the transmission initial wave could effectively characterize the stomata defect.

**Figure 18:**
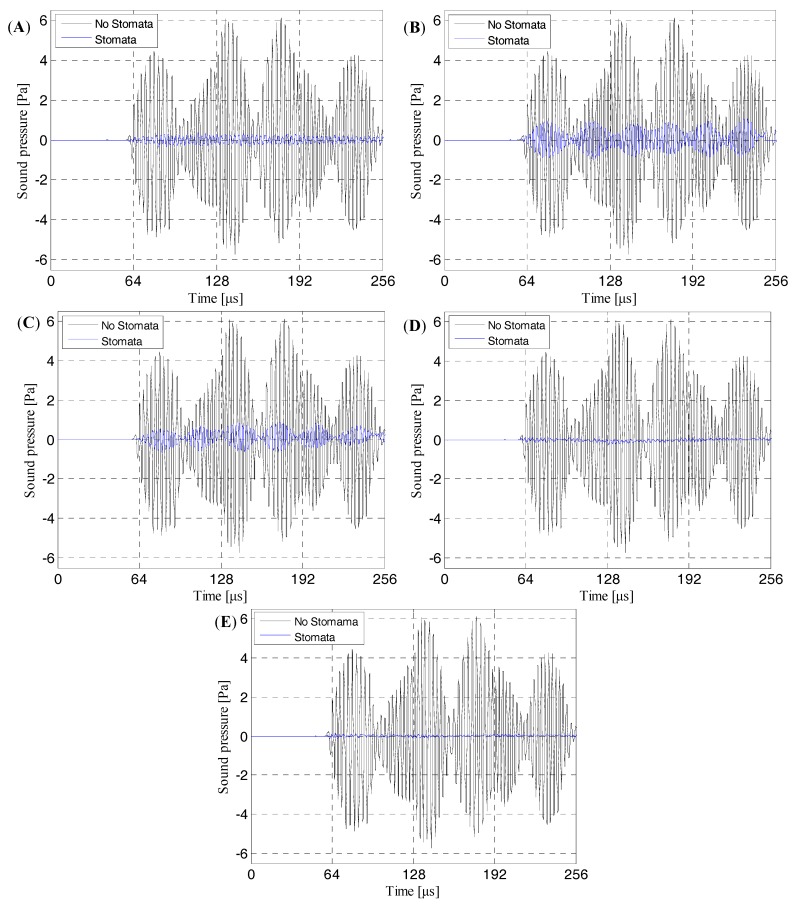
The transmission signals of the intact model and defective model with a stomata defect in the second, third, fourth, fifth and sixth electrolyte layer of the lithium-ion battery. (**A**) The stomata defect in the second electrolyte layer of the lithium-ion battery, (**B**) the stomata defect in the third electrolyte layer of the lithium-ion battery, (**C**) the stomata defect in the fourth electrolyte layer of the lithium-ion battery, (**D**) the stomata defect in the fifth electrolyte layer of the lithium-ion battery and (**E**) the stomata defect in the sixth electrolyte layer of the lithium-ion battery.

Figure 18 shows the contrast effect of the transmission ultrasonic signal received by the discrete probe points, corresponding to Figure 13, Figure 14, Figure 15, Figure 16 and Figure 17. As can be seen from Figure 18A, when there was a stomata defect in the second electrolyte layer of the lithium-ion model, the amplitude of the transmission initial signal would be significantly attenuated, and the energy of the transmission initial signal was reduced from 4.449 Pa with no defect to 0.261 Pa with a stomata defect. The same result could be obtained from Figure 18B–E: The amplitude of the transmission initial signal would be significantly attenuated, the energy of the transmission initial signal of Figure 18B attenuated to 0.944 Pa, the energy of the transmission initial signal of Figure 18C attenuated to 0.541 Pa, the energy of the transmission initial signal of Figure 18D attenuated to 0.143 Pa and the energy of the transmission initial signal Figure 18E attenuated to 0.151 Pa. Therefore, it could be confirmed that the stomata defects in the lithium-ion battery could be effectively characterized based on the change of the transmission initial wave.

In order to visually compare the energy attenuation of ultrasonic signal, the calculation formula of acoustic attenuation equivalent (9) was introduced:

(9) $$\Delta \;=20\mathrm{lg}\left(\frac{A_{2}}{A_{1}}\right)$$

where *A*_1_ is the amplitude of the transmission initial signal of the intact lithium-ion battery, *A*_2_ is the amplitude of the transmission initial signal of the defective lithium-ion battery. The calculation results are shown in Table 4.

The above simulation results show that the homogenized finite element model of the lithium-ion battery could correctly invert the sound wave propagation process in the lithium-ion battery; the receiver simulation transmission signal had a good signal to noise ratio (SNR) and can clearly identify the transmission initial wave (corresponding to the longitudinal wave in air, electrolyte and solid); the simulation transmission signal could effectively characterize the stomata defect in the first to sixth electrolyte layers; it is reasonable to use a transducer with a center frequency of 400 kHz as a receiving transducer in actual detection. Table 4 shows that when there were stomata defects in the third and fourth electrolyte layer, the acoustic attenuation equivalent was smaller than that in the first, second, fifth and sixth electrolyte layers with a stomata defect, and this was mainly due to the fact that the acoustic diffraction effect was more obvious when there was a stomata defect in the third and fourth electrolyte layer.

### 4.3. Transmission Signal in Actual Detection of the Lithium-Ion Battery

**Figure 19:**
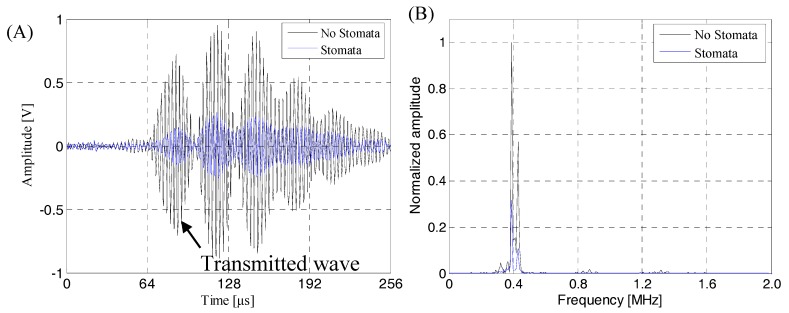
Air-coupled ultrasonic signal and its frequency spectrum during actual detection. (**A**) The transmission signal; (**B**) The corresponding frequency spectrum.

**Figure 20:**
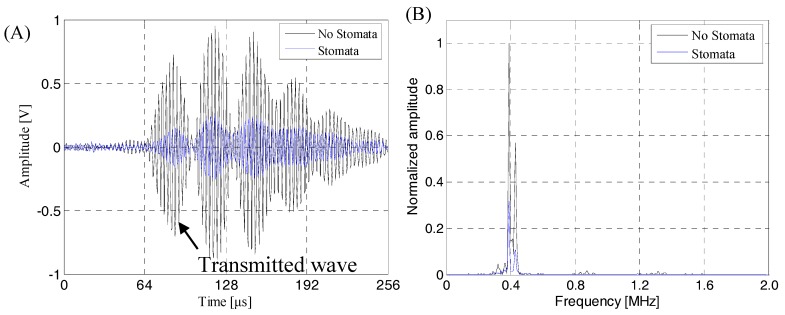
The contrast between the transmission signal of the near-surface stomata defect of a lithium-ion battery and the transmission signal of an intact lithium-ion battery sample. (**A**) The transmission signal; (**B**) The corresponding frequency spectrum.

**Figure 21:**
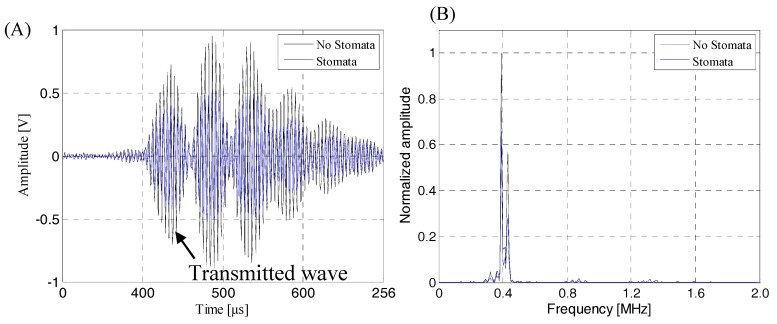
The contrast between the transmission signal of a natural stomata defect inside a lithium-ion battery and the transmission signal of an intact lithium-ion battery sample. (**A**) The transmission signal; (**B**) The corresponding frequency spectrum.

**Figure 22:**
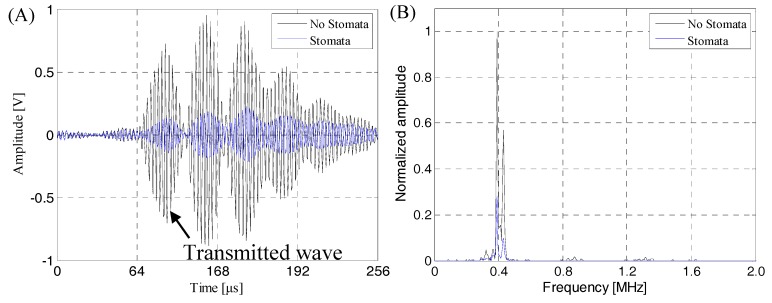
The contrast between the transmission signal of a near-bottom stomata defect of a lithium-ion battery and the transmission signal of an intact lithium-ion battery sample. (**A**) The transmission signal; (**B**) The corresponding frequency spectrum.

Combined with the lithium-ion battery finite element model and actual lithium-ion battery structure, we could see that the air layers set in the first electrolyte layer and the second electrolyte layer of the lithium-ion battery finite element model were used to simulate the near-surface stomata defect of the lithium-ion battery, the air layers set in the third electrolyte layer and the fourth electrolyte layer were used to simulate the middle-layer stomata defect of lithium-ion battery and the air layers set in the fifth electrolyte layer and the sixth electrolyte layer were used to simulate the near-bottom stomata defect of the lithium-ion battery. In order to verify the correctness of the finite element analysis result, a lithium-ion battery with near-surface defects, near-bottom defects and internal natural defects was prepared. The actual detection experiment was carried out. The results of the detection are shown in Figure 19, Figure 20, Figure 21 and Figure 22. The operating frequency of the air-coupled transducer was 400 kHz, the excitation voltage was 400 V, the gain was 60 dB and the excitation signal was a sine wave pulse train. Figure 19 shows the detection results of the intact lithium-ion battery sample. As can be seen from Figure 19A, the time domain signal had a good SNR and the transmission initial wave could be clearly identified. As can be seen from Figure 19B, the frequency-domain characteristics of the receiver signal were affected by various noise of the environment and ACUT system, resulting in narrowing of the frequency band, but the basic characteristics were not changed. The energy was mainly concentrated between 360 kHz and 440 kHz, and the signal energy reached the maximum value when the frequency of signal was 400 kHz.

Figure 20 shows the contrast between the transmission signal of the near-surface stomata defect of the lithium-ion battery and transmission signal of the intact lithium-ion battery sample. As can be seen from Figure 20A, the existence of the near-surface stomata defect could cause serious attenuation of the ultrasonic transmission signal, especially in the transmission initial wave. The amplitude of the transmission initial signal was reduced from 0.722 V with no defect to 0.148 V with a stomata defect, which was consistent with the finite simulation result. Seen from Figure 20B, the spectrum characteristic of the ultrasonic signal did not change regardless of the presence or absence of the near-surface stomata defect. The frequency band was narrower than the simulation result, but the basic characteristics were not changed, that is, the energy of transmission signal was mainly concentrated between 360 kHz and 440 kHz, and the signal energy reached the maximum value when the frequency of signal was 400 kHz. Therefore, it is reasonable to use a transducer with a center frequency of 400 kHz as a receiving transducer to evaluate the near-surface stomata defect of a lithium-ion battery during actual detection.

The same results can be obtained from Figure 21 and Figure 22: The amplitude of the transmission initial signal was reduced to 0.426 V with a natural stomata defect (see Figure 21A), the amplitude of transmission initial signal was reduced to 0.139 V with a near-bottom defect (see Figure 22A) and the spectrum characteristics of the transmission signal in Figure 21B and Figure 22B were identical to those presented in Figure 20B. The calculation results of attenuation were equivalent of the actual ultrasonic transmission signal for stomata defects at a different depth position of the lithium-ion battery as shown in Table 5.

The above test results were consistent with the finite element simulation results. The specific performance in the waveforms of the received signals were basically the same, that is, the received signals had good SNR and could clearly identify the transmission initial wave; the spectrum of the received transmission signals had good consistency regardless of the presence or absence of a stomata defect, that is, the transmission received signals had a narrow frequency band characteristic, energy mainly was concentrated between 360 kHz and 440 kHz, and the signal energy reached the maximum value when the frequency of signal was about 400 kHz; based on the transmission initial wave of the received signal, the response of the stomata defect at a different depth position was consistent, and the energy attenuation caused by the middle-layer stomata defect of lithium-ion battery was smaller than that of the near-surface stomata defect and near-surface stomata defect (Table 4 and Table 5 show the same regularity, but acoustic attenuation equivalent of the actual experimental results was larger than that of the finite element results. This was due to the fact that some factors leading to acoustic attenuation were idealized in the finite element simulation, so this was in line with the actual situation). Therefore, based on the correctness and consistency of the finite element simulation and the experimental results, it was proved that the stomata defect at different positions of the lithium-ion battery can be effectively characterized by the change of energy amplitude of the transmission initial wave of the transmission wave.

### 4.4. ACUT C-Scan of the Lithium-Ion Battery Sample

**Figure 23:**
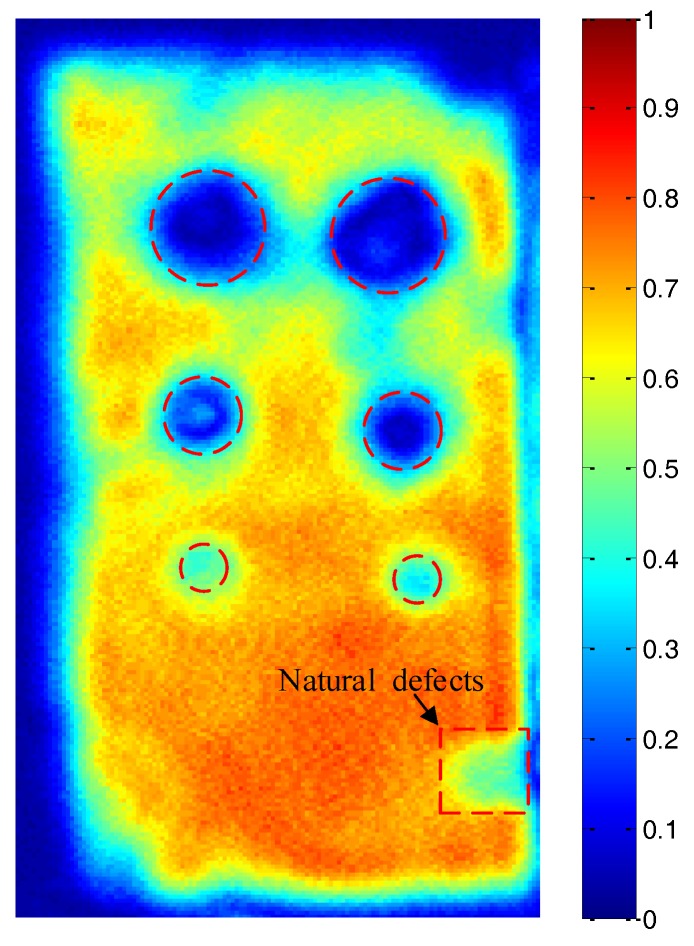
The C-scan results of the lithium-ion battery.

According to the above simulation result and experimental result, the stomata defect at a different depth position of the lithium-ion battery could be detected and characterized based on the transmission initial wave of the air-coupled transmission wave. Therefore, the ACUT C-scan image based on the signal in Figure 19A for the detection of the lithium-ion battery sample (mentioned in Section 3) could be achieved. The excitation voltage was 400 V, the total system gain was 60 dB, the scanning speed was 15 mm/s, the center frequency of the transducer was 400 kHz, the distance between the transducer, lithium-ion battery was 30 mm, the scanning area was 200 mm × 100 mm and the scanning step was 0.5 mm. The result is shown in Figure 23. It can be seen that the C-scan result was consistent with the actual features of the stomata defect in the lithium-ion battery as shown in Figure 8. At the same time, when the size of the stomata defect areas was similar, the overall energy of the natural defect was higher than that of the near-surface stomata defect and the near-bottom stomata defect (refer to the near-surface stomata defect with a size of Φ10 mm, the near-bottom stomata defect with a size of Φ10 mm and the natural stomata defect in Figure 23), which was consistent with the simulation result. Due to the influence of the focused sound beam, the overall energy of the small stomata defect was higher than the large stomata defect (refer to the near-surface stomata defect with a size of Φ20 mm, Φ10 mm and Φ5 mm or the near-bottom stomata defect with a size of Φ20 mm, Φ10 mm and Φ5 mm in Figure 23), which was consistent with the actual detection law of ACUT.

## 5. Conclusions

In this paper, aiming at the characteristics of the periodic stacking structure of a lithium-ion battery core and the corresponding relationship between the air-coupled ultrasonic transmission initial wave and the wave propagation mode in each medium layer of a lithium-ion battery, the homogenized finite element model of a lithium-ion battery was proposed for the first time. This model effectively solves the problem that the lithium-ion is difficult to be modeled because of the large number and the small size of dielectric layers. It provides a reliable method for correctly inverting the propagation of sound wave in each layer of the lithium-ion battery.

Based on the homogenization finite element model of a lithium-ion battery, the acoustic time-domain response and frequency-domain characteristic of the stomata defect at a different depth position of the lithium-ion battery were studied. The simulation results showed that the homogenization finite element model of the lithium-ion battery could correctly invert the sound wave propagation process in the lithium-ion battery; the simulation transmission signal had a good SNR and could clearly identify the transmission initial wave; the simulation transmission signal could effectively characterize the stomata defect in the first to sixth electrolyte layers, that is, when the stomata defect existed, the difference of the sound field was obvious, and the energy attenuation of the transmission sound wave was serious; the effect of the acoustic diffraction effect on the transmission signal energy was analyzed. Finally, the correctness and effectiveness of the homogeneous lithium-ion battery model was proved by practical experiments. At the same time, the agreement between the theoretical simulation results and the actual experimental results also proved that the stomata defect at different positions of the lithium-ion battery could be effectively characterized by the change of energy amplitude of the transmission initial wave of the transmission wave.

The ACUT C-scan image based on the signal in Figure 19A for the detection of the lithium-ion battery sample had been achieved. The C-scan result was consistent with the actual features of the stomata defect in the lithium-ion battery, which further demonstrates that ACUT technology is effective for the detection of the stomata defect in a lithium-ion battery. In the future, the research on detectability of lithium-ion battery types, defect characteristics of a lithium-ion battery will promote and expand the application of ACUT technology in the field of electric energy safety. 

## Figures and Tables

**Table 1:** Medium mesh size information.

Material	Acoustic Velocity/m/s	Mesh Size/μm
Air	340	100
PP_Al-plastic films	1900	30
Al_Al-plastic films	3080	30
nylon_Al-plastic films	1800	20
Electrolyte	1500	20
PP_diaphragm	1900	50
LiCoO2 coating	4057	50
Al_collector	3080	50
Graphite coating	3500	50
Cu_collector	3810	40

**Table 2:** The material properties of the fluid domain.

Fluid Medium	Density/kg/m^3^
Air	1.29
Electrolyte	1260

**Table 3:** The material properties of the solid domain.

Solid Medium	Elastic Modulus/GPa	Poisson Ratio	Density/kg/m^3^
LiCoO2	191	0.24	4790
Graphite	191	0.25	4790
Cu	119	0.326	8900
Al	71.7	0.33	2700
PP	0.896	0.4103	920
Nylon	2	0.4	1150

**Table 4:** The acoustic attenuation equivalent of the simulation ultrasonic transmission signal.

Defect Electrolyte Layer Location	Sound Pressure/Pa	Attenuation Equivalent/dB
No defect	4.449	0
First layer	0.126	−6.958
Second layer	0.261	−5.536
Third layer	0.944	−3.027
Fourth layer	0.541	−4.114
Fifth layer	0.143	−6.711
Sixth layer	0.151	−6.605

**Table 5:** The acoustic attenuation equivalent of the actual ultrasonic transmission signal.

Defect Electrolyte Layer Location	Amplitude/V	Attenuation Equivalent/dB
No defect	0.722	0
Near-surface layer	0.148	−19.066
Middle layer	0.426	−6.347
Near-bottom layer	0.139	−19.821
